# The Effect of Transcutaneous Vagus Nerve Stimulation in Patients with Polymyalgia Rheumatica

**DOI:** 10.3390/ph14111166

**Published:** 2021-11-16

**Authors:** Jacob Venborg, Anne-Marie Wegeberg, Salome Kristensen, Birgitte Brock, Christina Brock, Mogens Pfeiffer-Jensen

**Affiliations:** 1 Department of Rheumatology, Aarhus University Hospital, 8200 Aarhus, Denmark; jacob.venborg@clin.au.dk; 2 Mech-Sense, Department of Gastroenterology and Hepatology, Aalborg University Hospital, 9000 Aalborg, Denmark; a.wegeberg@rn.dk (A.-M.W.); christina.brock@rn.dk (C.B.); 3 Department of Rheumatology, Aalborg University Hospital, 9000 Aalborg, Denmark; sakr@rn.dk; 4 Department of Clinical Medicine, Aalborg University, 9000 Aalborg, Denmark; 5 Steno Diabetes Center Copenhagen, 2820 Gentofte, Denmark; birgitte.brock@regionh.dk; 6 Department of Clinical Medicine, Faculty of Health Sciences, University of Copenhagen, 2200 Copenhagen, Denmark; 7 Copenhagen Center for Arthritis Research (COPECARE), Center for Rheumatology and Spine Diseases, Rigshospitalet, 2600 Glostrup, Denmark

**Keywords:** polymyalgia rheumatica, vagus nerve stimulation, inflammatory response, PMR, t-vns

## Abstract

(1) Polymyalgia rheumatica (PMR) is an inflammatory disease characterised by pain, morning stiffness, and reduced quality of life. Recently, vagus nerve stimulation (VNS) was shown to have anti-inflammatory effects. We aimed to examine the effect of transcutaneous VNS (t-VNS) on PMR. (2) Fifteen treatment-naïve PMR patients completed the study. Patients underwent a 5-day protocol, receiving 2 min of t-VNS stimulation bilaterally on the neck, three times daily. Cardiac vagal tone (CVT) measured on a linear vagal scale (LVS), blood pressure, heart rate, patient-reported outcome, and biochemical changes were assessed. (3) t-VNS induced a 22% increase in CVT at 20 min after initial stimulations compared with baseline (3.4 ± 2.2 LVS vs. 4.1 ± 2.9 LVS, *p* = 0.02) and was accompanied by a 4 BPM reduction in heart rate (73 ± 11 BPM vs. 69 ± 9, *p* < 0.01). No long-term effects were observed. Furthermore, t-VNS induced a 14% reduction in the VAS score for the hips at day 5 compared with the baseline (5.1 ± 2.8 vs. 4.4 ± 2.8, *p* = 0.04). No changes in CRP or proinflammatory analytes were observed. (4) t-VNS modulates the autonomic nervous system in patients with PMR, but further investigation of t-VNS in PMR patients is warranted.

## 1. Introduction

Polymyalgia rheumatica (PMR) is an inflammatory rheumatic disease of unknown aetiology characterised by muscle pain and morning stiffness in the shoulders, pelvic girdle, and neck. PMR is rarely seen in persons below the age of 50, and can occur independently or alongside giant cell arteritis [1,2]. Typically, PMR will “burn out” after approximately 2 years and it is not associated with increased mortality [3,4,5]. Nevertheless, ordinary daily activities become immensely difficult and painful to accomplish; consequently, PMR patients often describe a great decline in quality of life. Finally, the disease is associated with increased usage of primary healthcare [6], and thus effective treatment restoring quality of life is of paramount importance to patients with PMR and their families. The first-choice treatment is systemically administered low doses (initially 12.5–25 mg/day) corticosteroids [7]. Although this treatment provides quick and efficient recovery, the adverse effects are typically numerous and severe [8]. Among these, osteoporosis, skin thinning, cushingoid appearance, weight gain, myopathy, and mood disorders are common [9]; as such, effective treatments with less negative and unwanted side effects are warranted. Biological anti-inflammatory drugs are frequently used within rheumatology. Recent studies have demonstrated that PMR patients had increased serum levels of interleukin 6 (IL-6) in comparison with healthy controls, suggesting that IL-6 was part of the pathogenesis of PMR [10,11]. This is supported by the efficient use of monotherapy with IL-6 inhibitors in PMR patients [12,13,14,15]. However, treatment with biological drugs is still associated with side effects, such as the increased the risk of infection, fever, and rash.

It is generally accepted that the autonomic nervous system regulates neuro-immune communication primarily through the vagal nerve. In vitro studies have shown the inhibition of macrophage cytokine release in lipopolysaccharide-stimulated human macrophage cultures enriched with the cholinergic neurotransmitter acetylcholine [16]. Moreover, direct electrical stimulation of the vagus nerve in rats diminished serum levels of tumour necrosis factor-alpha (TNF-α) [17]. Vagal nerve stimulation (VNS) is also believed to diminish levels of pro-inflammatory cytokines, such as IL-1 and IL-6, the latter of which is of great interest in PMR patients [18]. In studies of healthy humans, transcutaneous vagus nerve stimulation (t-VNS) was shown to modulate the inflammatory response by increasing the cardiac vagal tone (CVT) and decreasing the systemic level of TNF-α [16,19]. Finally, t-VNS has reduced disease activity scores in patients with well-controlled psoriatic arthritis (PsA) and rheumatoid arthritis (RA) with no reported adverse effects [20,21]. However, a knowledge gap remains, as no studies have previously investigated the effect of t-VNS as an exclusive treatment in treatment-naïve patients with diseases characterised by high-grade inflammation.

Thus, we aimed to investigate the effect of 5-day t-VNS in treatment-naïve patients with PMR. We hypothesised that t-VNS would increase CVT and consequently reduce the inflammatory response, leading to clinical improvement in patients with PMR. Thus, the aims of this proof-of-concept study were to assess (1) the acute and 5-day CVT response to t-VNS; (2) the effect of 5-day t-VNS on cardiac-derived parameters, such as blood pressure (BP) and heart rate (HR); (3) the effect of t-VNS on inflammatory biomarkers; and (4) patient-reported inflammatory pain.

## 2. Results

Fifteen of the twenty enrolled patients completed the study. The baseline characteristics of the population are shown in Table 1. The intention-to-treat approach was used, and due to the investigation of various parameters, some datapoints may be missing in a subgroup of patients either because they were extreme values or because the assays were performed incorrectly. Consequently, such values were excluded from further analyses. No adverse events were reported. On average, each patient received 24 stimulations, which means they received fewer than planned (26).

### 2.1. Changes in Primary Outcome: Cardiac Vagal Tone

One patient had faulty CVT recordings at all visits; consequently, these measurements were excluded. Another patient showed an extreme value of CVT on day 2; thus, this single measurement was excluded. Only measurements of CVT were excluded; the other parameters were not.

An acute 22% increase in CVT was observed 20 min after the initial t-VNS (3.4 ± 2.2 LVS vs. 4.1 ± 2.9 LVS, *p* = 0.02). However, no changes in CVT were observed on day 2 (3.4 ± 2.2 LVS vs. 3.9 ± 2.7 LVS, *p* = 0.50) nor on day 5 (3.4 ± 2.2 LVS vs. 4.2 ± 2.9, *p* = 0.20). The results are shown in Table 2.

### 2.2. Changes in Secondary Outcomes

#### 2.2.1. Changes in Cardiac-Derived Parameters

**Figure 1:**
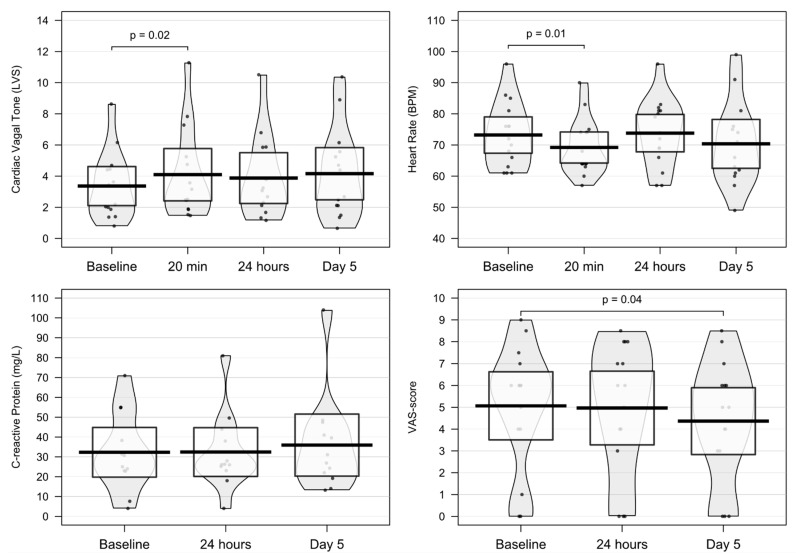
Raw data points, mean, and 95% CI of selected outcomes.

An acute decrease of 4 BPM in resting HR was observed 20 min after initial t-VNS (73 ± 11 BPM vs. 69 ± 9, *p* < 0.01). No changes in resting HR were observed on day 2 (73 ± 11 BPM vs. 74 ± 11 BPM, *p* = 0.77) or on day 5 (73 ± 11 BPM vs. 70 ± 14, *p* = 0.27). No changes in systolic or diastolic BP were observed 20 min after initial t-VNS, on day 2, or on day 5. The results are shown in Table 2 and Figure 1.

#### 2.2.2. Changes in CRP and Proinflammatory Analytes

Two patients were diagnosed obs. pro PMR but had no concomitant increase in markers of CRP; consequently, they were excluded from the analysis of changes in CRP. Furthermore, a single patient showed extreme values for CRP due to an infection and was excluded for analysis.

No changes in CRP were observed in response to t-VNS on day 2 (32.3 ± 19.7 mg/L vs. 32.4 ± 19.3, *p* = 0.94) or on day 5 (32.3 ± 19.7 mg/L vs. 35.9 ± 24.6 mg/L, *p* = 0.33) in comparison with the baseline. The results are shown in Table 2 and Figure 1. No changes were observed in any of the investigated analytes.

#### 2.2.3. Changes in Patient-Reported Outcome

A 14% reduction in the VAS score for the hips was shown on day 5 in comparison with baseline (5.1 ± 2.8 vs. 4.4 ± 2.8, *p* < 0.05). No significant changes were observed in MHAQ scores, VAS score of PMR influence, global VAS score, or duration of morning stiffness on day 2 or on day 5. The results are shown in Table 2 and Figure 1.

## 3. Discussion

To our knowledge, this is the first report of response to t-VNS in patients with PMR. We demonstrated that t-VNS caused an acute increase in CVT alongside a decrease in HR in treatment-naïve patients with PMR as a response to bilateral stimulation, indicating that an acute modulation of the autonomic nervous system was obtained. Furthermore, we demonstrated pain relief related to the hips in response to t-VNS.

In this study, we found lower mean values of CVT at baseline and on day 5 when compared with healthy individuals of similar age [22]. However, although this is the first preliminary report on CVT values in patients with PMR, it is consistent with low CVT values in patients with chronic pancreatitis and diabetes mellitus type 1 [23,24]. Furthermore, impaired parasympathetic activity has been demonstrated in patients with Crohn’s disease [25]. Thus, our findings seem to support that the autonomic nervous system regulates neuro-immune communication and the activation of the cholinergic anti-inflammatory reflex. A true increase in CVT following 5 days of stimulation may exist; however, this result may subsequently be hampered by the presence of a type 2 error due to low power.

PMR is a clinical diagnosis that may be difficult to establish with certainty due to the heterogeneity of the disease [26]. In 2012, The European League Against Rheumatism (EULAR) in collaboration with the American College of Rheumatology (ACR) developed provisional classification criteria for PMR in order to make the process of diagnosis more consistent [27]. However, these criteria are not meant for diagnostic purposes. Patients were eligible for inclusion if the doctor’s putative diagnosis was PMR or obs. pro. PMR, and thus, the diagnoses were not definitive at the point of enrolment.

We observed a 14% decrease in VAS score of the pain related to the hips, and similar trends were shown for the rest of the parameters. This suggests that t-VNS might reduce pain. Furthermore, although PMR is not associated with increased mortality, patients with PMR typically describe an immense loss in quality of life. To evaluate the patient’s self-reported function and quality of life, MHAQ score, VAS scores, and duration of morning stiffness were evaluated but were not altered in response to t-VNS.

We did not observe any decrease in the objective biochemical profile, including CRP and IL-6, which are of particular interest in PMR. This contrasts with findings in other inflammatory diseases, such as rheumatoid and psoriatic arthritis, where t-VNS resulted in decreased levels of CRP [21]. These contrasting findings may be due to the high-grade inflammation in PMR patients. Koopman et al. demonstrated inhibited levels of TNF-α and improved disease scores in response to VNS stimulation for 42 days with an implanted device in patients with RA, suggesting the long-term activation of the cholinergic anti-inflammatory reflex [28]. In contrast, we applied stimulation three times per day over 5 days with a handheld, non-invasive device, and the treatment was mostly patient-administered. Thus, this study might underestimate the potential benefit of VNS on pro- and anti-inflammatory cytokines in PMR.

### Limitations

This study is the first of its kind to investigate the effect of transcutaneous vagal nerve stimulation in patients with PMR characterised by high-grade inflammation. It is, however, an open-label, proof-of-concept study and thus has inherent limitations. First, as we did not have a sham control group, we cannot make firm conclusions on the observed changes in response to VNS. Second, as the study was open-label, all patients were aware of any beneficial effect and may have influenced subjective outcomes. However, all patients were examined by the same two researchers; thus, instructions on how to use the gammaCore were standardised, which minimised the risk of difference in the quality of the stimulation. Third, the study may be underpowered as we aimed to include 20 patients, but only 15 completed the protocol. Therefore, the sample size was small and vulnerable to inducing error. However, similar explorative pilot studies have been able to show differences in response to t-VNS in patient groups with established rheumatoid diagnoses [20,21]. Fourth, the intervention length of 5 days may have been insufficient for t-VNS to alter the disease activity. However, as these patients were treatment-naïve and suffered from pain, we did not believe it ethical to prolong this explorative treatment. Consequently, all patients in the study went on to be treated with prednisolone. Nonetheless, we cannot rule out the possibility that a longer intervention might have produced pronounced effects. Fifth, it has been questioned whether 5 min CVT was a reliable biomarker of parasympathetic activation, but the measure has been shown to perform better than heart rate variability measures [29]. Lastly, with only three out of the total 26 stimulations being supervised, we could not ensure the quality of each stimulation.

## 4. Materials and Methods

### 4.1. Study Design

This study was an open-label, proof-of-concept experimental pilot study investigating the effect of t-VNS in patients with inflammatory diseases. Two centres were used for inclusion: Mech-Sense, Aalborg University Hospital, and Department of Rheumatology, Aarhus University Hospital. A 5-day protocol was used, which was believed to be of adequate length to demonstrate our hypothesis, but did not unnecessarily delay treatment with glucocorticoids.

### 4.2. Cohort

Forty-two patients did not meet any exclusion criteria and were eligible for screening by a trained doctor to confirm the diagnosis of either (1) a well-established diagnosis of PMR (certain diagnosis) or (2) a putative PMR diagnosis, where no alternative pathology could explain the case better (obs. pro PMR). Exclusion criteria were any corticosteroid treatment within 5 weeks prior to inclusion, age < 18 years, known cardiovascular disease, hypotension (<100 mmHg systolic and <60 mmHg diastolic), pregnancy (positive U-HCG) or current lactation, and non-compliance with the protocol. Inclusion criteria were newly diagnosed, treatment-naïve PMR patients. No drugs were used besides NSAIDs and paracetamol; while the usage of NSAIDs during the protocol was not disallowed, it was recommended that patients did not use it.

If the eligible patients agreed to participate, they signed an informed consent form. Twenty patients were included; however, 5 patients dropped out before completion, leaving 15 patients for analysis. Reasons for drop-out were: non-compliance with the protocol (n = 1), withdrawal of consent (n = 2), and did not show (n = 2).

### 4.3. Vagus Nerve Stimulation

t-VNS was performed using a non-invasive, handheld gammaCore^®^ device (electroCore, Inc., Basking Ridge, NJ, USA) providing transcutaneous low-voltage electric stimulation on the cervical part of the vagus nerve. The signal consisted of five 5000 Hz sine-wave pulses repeated at a rate of 25 Hz. Patients were given clear instructions to place the two gel-covered conductors on top of the common carotid arteries on the neck. The amplitude of the electric signal, ranging from 0 to 40 on an arbitrary scale, could be adjusted via two control buttons on the device. Each stimulation lasted 2 min, after which the device would stop automatically.

On days 1–4, stimulations were carried out bilaterally three times a day (morning, noon, and evening), while on day 5 only one stimulation was carried out. A total of 26 stimulations were planned for each patient. Compliance was assured by counting the remaining stimulations when the device was returned. The patients were given clear instructions to position the device correctly, and the amplitude was to be slowly increased until a mild contraction of the ipsilateral oral commissure was seen or the pain from the stimulation was unbearable. At the second visit, the patients performed a stimulation under the supervision of the investigator to ensure safe and proper usage.

### 4.4. Outcomes

#### 4.4.1. Primary Outcome: Resting Cardiac Vagal Tone

The primary outcome was a change in resting CVT between baseline and day 5 (long-term response) and differences between baseline and 20 min after the first stimulation (acute response).

CVT is a non-invasive measure of the efferent parasympathetic cardiac vagal tone, which is computed from a five-minute ECG recording; incoming QRS complexes are compared with a template derived from the initial part of the recording, and changes in R–R intervals are detected via phase shift demodulation [22]. CVT was measured on a linear vagal scale where 0 represents full atropinisation [30]. Resting CVT was assessed via a three-lead ECG (eMotion Faros180° portable cardiac monitoring device, Bittium, Oulu, Finland) using Ambu BlueSensor P ECG-electrodes (Ambu, Copenhagen, Denmark), placed on cleaned and dried skin, and assessments were performed in conformity with international recommendations [31]. The recordings were analysed using ProCVT software (ProBiometrics, London, UK) to derive CVT.

On days 1, 2, and 5, five-minute ECG recordings were conducted. On day 1, two recordings were made to evaluate the acute response; one prior to the first stimulation (baseline) and the second after 20 min. On days 2 and 5, a single CVT recording was performed before stimulation. The successful recordings were manually edited if needed, i.e., changes in HR exceeding 15 beats per minute (BPM) between two consecutive heartbeats were treated as artefacts, e.g., coughing or sudden movements. If artefacts were present in the data, the five heartbeats before and after were discarded by the underlying algorithm.

#### 4.4.2. Secondary Outcomes: Cardiac-Derived Parameters, CRP, Proinflammatory Analytes, and Patient-Reported Outcome

Patient-reported outcomes were assessed on days 1, 2, and 5, with each patient completing two questionnaires. Firstly, the modified health assessment questionnaire (MHAQ), which consists of eight questions measuring the ability to perform common daily life activities, such as dressing, arising, eating, walking, hygiene, reaching, and gripping. Each patient was asked to rate their ability to perform these activities on a scale ranging from 1 to 4: 1 = without difficulty, 2 = with some difficulty, 3 = with much difficulty, and 4 = unable to do the requested task. The second questionnaire consisted of three assessments on a validated continuous (0–100 mm) visual analogue scale (VAS scoring) and an evaluation of the duration of morning joint stiffness. For the VAS score, three domains were assessed: (1) general pain, (2) pain related to the hips, and (3) a general, overall assessment of the negative effect and influences caused by PMR.

Measurement of BP and HR was carried out prior to each ECG recording. Each measurement was performed on the upper left arm using an electronic sphygmomanometer (UA-852; A&D Company, Limited, Tokyo, Japan).

Blood samples were drawn on days 1, 2, and 5 prior to other measurements. Samples for routine clinical biochemistry, alongside EDTA-plasma and serum, were drawn on baseline day and day 5 for analysis of proinflammatory analytes IFN-γ, IL-,1β, IL-2, IL-4, IL-6, IL-8, IL-10, IL-13, and TNF-α. Analyses of cytokines were performed via Luminex multiplexing technology using the Inflammation 20-Plex Human ProcartaPlex™ Panel (Invitrogen, Thermo Fisher Scientific, Waltham, MA, USA) and a MAGPIX instrument (Luminex, Austin, TX, USA) in accordance with the manufacturer’s protocol. For each analyte, extreme outliers, defined as values above Q3 + 3 × IQR or below Q1 − 3 × IQR, were identified and removed.

### 4.5. Statistical Methods

Data were presented as mean ± standard deviation (SD) unless otherwise clarified. All data were evaluated for normality using Shapiro–Wilk test for normality or through visual inspection of QQ plots. For statistical comparison between baseline and visit values, paired *t*-test was used for data of normal distribution, and Wilcoxon signed-rank test was used for data of non-normal distribution. A *p*-value less than 0.05 was considered significant. All data analyses were performed in STATA version 16.0 (StataCorp, TX, USA) and R version 4.0.3 (The R Foundation for Statistical Computing). Likewise, all graphical outputs were produced using the same version of R.

## 5. Conclusions

In conclusion, we showed an acute modulation of the autonomic nervous system in patients with PMR as evidenced by increased CVT and decreased HR. Furthermore, we showed alleviation of hip pain in response to a five-day protocol, but this was not reflected in the cytokine profile. Further investigation of t-VNS in PMR patients is warranted, preferably in blinded, randomised, sham-controlled trials, before any firm conclusion is drawn upon the ability to activate the cholinergic anti-inflammatory reflex in this patient group.

## Figures and Tables

**Table 1:** Demographic and General Population Characteristics.

Characteristic	PMR Patients (n = 15)
Sex (female)	13 (87)
Age (years)	65 ± 10
Height (cm)	169 ± 6
Weight (kg)	72 ± 12
Body mass index (kg/m^2^)	25 ± 4
Currently using NSAIDs (yes)	6 (40.0)
Daily NSAID dose (mg ibuprofen)	833 ± 480
Ethnicity (Caucasian)	15 (100.0)
Smoking, ever (yes)	7 (47)
Smoking (pack-years)	16 ± 12
Daily caffeine intake (yes)	15 (100)
Stimulations pr. patient (mean out of 26)	24 (91)
Amplitude of baseline stimulation	33 ± 6

Data are given as mean ± SD or no. (%) unless stated otherwise.

**Table 2:** Changes in Outcomes.

	Baseline	20 min	24 h	Day 5	*p*-Value
Cardiac vagal tone (LVS)	3.4 ± 2.2	4.1 ± 2.9	3.9 ± 2.7	4.2 ± 2.9	0.02 *
Systolic blood pressure (mmHG)	139 ± 22	141 ± 22	135 ± 19	137 ± 24	0.38
Diastolic blood pressure (mmHG)	79 ± 10	81 ± 10	77 ± 8	82 ± 15	0.53
Heart rate (BPM)	73 ± 11	69 ± 9	74 ± 11	70 ± 14	0.01 *
MHAQ score	0.9 ± 0.5	-	0.9 ± 0.5	0.8 ± 0.5	0.19
VAS score of PMR-influence	6.7 ± 2.6	-	6.4 ± 2.6	6.1 ± 2.5	0.23
VAS score in hips	5.1 ± 2.8	-	5.0 ± 3.1	4.4 ± 2.8	0.04
Global VAS score	6.2 ± 2.8	-	6.1 ± 2.7	5.9 ± 2.5	0.54
Duration of morning stiffness (minutes)	124 ± 89	-	120 ± 79	108 ± 65	0.19
C-reactive protein (mg/L)	32.3 ± 19.7	-	32.4 ± 19.3	35.9 ± 24.6	0.74
IFN-γ (pg/mL)	5.40 ± 2.67	-	-	6.20 ± 5.94	0.29
IL-2 (pg/mL)	0.06 (0.10)	-	-	0.12 (0.24)	0.06
IL-4 (pg/mL)	0.01 ± 0.01	-	-	0.03 ± 0.03	0.82
IL-6 (ng/L)	4.81 (4.80)	-	-	4.50 (6.25)	0.19
IL-8 (pg/mL)	12.72 ± 6.58	-	-	12.68 ± 6.90	0.37
IL-10 (pg/mL)	0.27 (0.14)	-	-	0.32 (0.12)	0.91
TNF-α (pg/mL)	1.35 ± 0.43	-	-	1.32 ± 0.44	0.67

Data are given as mean ± SD or median (interquartile range) unless otherwise stated. The *p*-values are a comparison between baseline and day 5. * Comparison between baseline and 20 min.

## Data Availability

Data is contained within the article.
